# Resuscitation on the pitch

**DOI:** 10.1007/s12471-017-1043-7

**Published:** 2017-10-10

**Authors:** N. M. Panhuyzen-Goedkoop, J. J. Piek

**Affiliations:** 1 Heart Center, Academic Medical Centre Amsterdam, Amsterdam, The Netherlands; 2 Sports Medical Centre Papendal, Arnhem, The Netherlands; 3 Heart Center, Radboudumc Nijmegen, The Netherlands

When an athlete dies suddenly this is a very tragic event, raising media attention and a discussion on how to protect athletes. As a result more pre-participation screening is requested to identify athletes with very rare inherited cardiovascular disorders at risk of the life-threatening cardiac arrhythmia i.e. ventricular tachycardia/fibrillation (VT/VF) [1]. The incidence of sudden cardiac death (SCD) in athletes is low (0.6–2.85/100,000 annually) [2, 3]. The annual incidences of SCD (3–10.7/100,000) and VT/VF (84.0/100,000) in the overall population are reported to be substantially higher [4, 5]. There are no comprehensive data on the incidence of out-of-hospital cardiac arrest in athletes [6]. Pre-participation cardiovascular screening in athletes is a widely accepted method for primary prevention of SCD. Including an ECG is still a topic of debate. However, there is strong support to include an ECG routinely in pre-participation cardiovascular screening in both young and master athletes [7]. If pre-participation screening fails, bystander resuscitation using an automatic external defibrillator (AED) is used for secondary prevention to save an athlete’s life [1].

In 2015 the European Resuscitation Council (ERC) guidelines for resuscitation (2010) were updated [8]. The most important updated issues are an interaction of emergency medical services with the bystander performing cardiopulmonary resuscitation (CPR) and the use of an automated external defibrillator (AED) [8]. The emergency medical services provide telephone assistance in CPR and the location of an AED [8]. Bystanders should be trained and able to assess rapidly if the victim is 1) unresponsive and 2) not breathing normally by checking the Airway (A) and Breathing (B). These key observations are critical in early recognition of Circulation arrest (C). Critical seconds can be lost when this sudden circulatory arrest (SCA) situation is not recognised immediately and time can be wasted trying to get access to and open the airway system [9]. In SCA (unresponsiveness and not breathing normally) CPR should be initiated immediately with chest compressions (depth 5–6 cm, rate 100–120 compressions/min) combined with rescue breaths (ratio 30:2) [8]. Public access AED programs are essential for early defibrillation. However, the ERC guidelines do not describe CPR in athletes and recreational sports participants.

When an athlete suddenly and unexpectedly collapses at a training session or during a match the most obvious cause is an underlying cardiac disorder [2, 3]. It is very important that such a life-threatening situation (VT/VF) is recognised early and CPR is commenced to save the athlete’s life [8]. Any witness of this SCA – i. e. medical personnel, team player, referee, spectator, or other person – should not hesitate and start chest compressions immediately as described by the ERC [8]. A second person is urged to fetch an AED if available at the sport’s facility and connects the AED to the victim without ceasing chest compressions. A third person should call 112 (in European countries) asking to send for an ambulance to assist CPR. In the meantime, putting your mobile phone on the loudspeaker, 112 will guide you through the resuscitation procedure until the ambulance personnel has arrived. When the AED says ‘rhythm analysis’ the chest compressions are briefly interrupted. Next the AED says ‘do not touch the patient’ and delivers an electrical shock in case of VT/VF. Thereafter, chest compressions should be continued immediately and uninterrupted (100/min) for two minutes. After these two minutes the AED will again perform a rhythm analysis and deliver an electrical shock when appropriate. This cycle of chest compressions (2 min) – AED – electrical shock – chest compressions (2 min) – AED – electrical shock – etc. will be repeated until the ambulance personnel have arrived and continue CPR. During CPR the airway (A) and breathing (B) of the victim can be analysed without ceasing chest compressions (C). Every second counts, and every second that chest compressions are not performed reduces the chance of survival. By starting chest compressions (C) immediately without any hesitation, the old and well-known rule ABC, i. e. Airway – Breathing – Circulation, as taught during the courses of basic life support (BLS) and advanced cardiac life support (ALS) and described in the ERC guidelines is overruled in Circulation first (CAB or CBA) [8, 9]. The chance that the cause of an athlete’s collapse during exercise is cardiac is almost 100% and the likelihood that this collapse is due to an airway obstruction is very small.

The success of bystander resuscitation in athletes on the pitch depends on witnessed and early recognition of SCA, willingness to resuscitate (cardiac compressions), call for help (112) and early defibrillation (AED) [6, 8]. The mean survival of SCA with bystander resuscitation before the ambulance has arrived in the overall population the Netherlands is 23% [10]. This survival rate is even higher if there is a shockable arrhythmia (VT/VF) initially (44%) [8]. In athletes there are unfortunately no data, but there is a need for a uniform registration of SCA in sports [6].

In conclusion, always chest compressions and AED first when an athlete collapses on the pitch because of an almost always cardiac origin of the event. Early recognition, cardiac compressions and early defibrillation are essential to survive SCA. The ERC is challenged to describe new and adequate CPR guidelines in athletes collapsing on the pitch taking into account the athlete’s high adrenergic state as well as his/her condition of volume depletion and electrolyte disturbance.

The Netherlands Heart Journal intends to publish a Special Issue on Sports Cardiology in Spring 2018 to discuss cardiac issues in athletes.
